# Radiographic and clinical outcomes of robot-assisted pedicle screw instrumentation for adolescent idiopathic scoliosis

**DOI:** 10.3389/fsurg.2024.1344802

**Published:** 2024-04-22

**Authors:** Yuan-Shao Chen, Yu-Hsien Lin, Yun-Che Wu, Cheng-Min Shih, Kun-Huei Chen, Cheng-Hung Lee, Wen-Hsien Lu, Chien-Chou Pan

**Affiliations:** ^1^ Department of Orthopedic Surgery, Taichung Veterans General Hospital, Taichung, Taiwan; ^2^ College of Medicine, National Chung Hsing University, Taichung, Taiwan; ^3^ Department of Physical Therapy, Hung Kuang University, Taichung, Taiwan; ^4^ Office of Research and Development, National Chung Hsing University, Taichung, Taiwan; ^5^ Department of Computer Science and Information Engineering, Providence University, Taichung, Taiwan; ^6^ Department of Food Science and Technology, Hung Kuang University, Taichung, Taiwan; ^7^ Department of Orthopedic Surgery, Feng Yuan Hospital, Ministry of Health and Welfare, Taichung, Taiwan; ^8^ Department of Rehabilitation Science, Jenteh Junior College of Medicine, Nursing and Management, Miaoli, Taiwan

**Keywords:** robot-assisted, pedicle screw instrumentation, adolescent idiopathic scoliosis, AIS, radiographic, clinical, outcome

## Abstract

**Introduction:**

Pedicle screw instrumentation (PSI) serves as the widely accepted surgical treatment for adolescent idiopathic scoliosis (AIS). The accuracy of screw positioning has remarkably improved with robotic assistance. Nonetheless, its impact on radiographic and clinical outcomes remains unexplored. This study aimed to investigate the radiographic and clinical outcomes of robot-assisted PSI vs. conventional freehand method in AIS patients.

**Methods:**

Data of AIS patients who underwent PSI with all pedicle screws between April 2013 and March 2022 were included and retrospectively analyzed; those with hybrid implants were excluded. Recruited individuals were divided into the Robot-assisted or Freehand group according to the technique used. Radiographic parameters and clinical outcome measures were documented.

**Results:**

In total, 50 patients (19, Freehand group; 31, Robot-assisted group) were eligible, with an average age and follow-up period of 17.6 years and 60.2 months, respectively, and female predominance (40/50, 80.0%). The correction rates of Cobb's angles for both groups were significant postoperatively. Compared to freehand, the robot-assisted technique achieved a significantly reduced breech rate and provided better trunk shift and radiographic shoulder height correction with preserved lumbar lordosis, resulting in significantly improved visual analog scale scores for back pain from the third postoperative month.

**Conclusion:**

Overall, robot-assisted PSI provides satisfactory radiographic and clinical outcomes in AIS patients.

## 1 Introduction

Scoliosis, defined as abnormal spinal rotation with a coronal curve greater than 10°, can be classified into three subtypes: congenital, neuromuscular, or idiopathic. Approximately 85% of cases are idiopathic, further categorized by age of onset: infantile (≤2 years), juvenile (3–9 years), and adolescent (≥10 years) (1). Adolescent idiopathic scoliosis (AIS) is the most common form, affecting 1%–3% of children aged 10–16 years (2). Approximately 10% of affected individuals progress and require surgical intervention (3). Surgery is indicated in individuals with a primary curve greater than a Cobb's angle of 45° (2). Correction of spinal deformity is crucial for improving a patient's health-related quality of life. Pedicle screw-only construct is safe, effective and reliable in correcting spinal deformities (4–6) and is still considered the widely accepted surgical option. However, screw implantation is particularly challenging if the patient presents with a higher degree of deformity or hypoplasia of the spinal pedicle (7). Spinal robotic technology offers a solution to this problem (8, 9). The Mazor Robotics's SpineAssist™ became the first FDA-approved robot to guide the placement of pedicle screws in 2004 (10). The accuracy of pedicle screw implantation has been well studied (8, 11, 12). However, there is a paucity of information on the radiographic and clinical outcomes, especially in patients with AIS. This study aimed to investigate the radiographic and clinical outcomes of robot-assisted (RO) pedicle screw instrumentation for AIS compared to those of the conventional freehand (FH) method.

## 2 Materials and methods

The study was conducted in accordance with the Declaration of Helsinki, and approved by the Institutional Review Board of our institution (No. CE21251B, date of approval: Jul 28, 2021). The requirement for written informed consent was waived by the institutional review board.

### 2.1 Study design

Considering our study's aim, we hypothesized that surgery using the RO technique approach would outperform that using the FH method regarding radiographic and clinical outcomes. To investigate this hypothesis, we consecutively enrolled 50 AIS patients who underwent all-pedicle-screw posterior instrumentation surgeries between April 2013 and March 2022 and analyzed their data. Medical records and radiographic images were retrospectively reviewed. Individuals were recruited according to the following criteria: (1) minimal follow-up period of one year, (2) availability of pre- and postoperative images, (3) all-pedicle-screw construct only, and (4) primary surgery. The exclusion criteria included: (1) congenital vertebral malformation (e.g., hemivertebra, spina bifida) and neuromuscular abnormalities, (2) spinal anchors other than pedicle screws (e.g., hook, clamp, wire), and (3) revision surgery.

The recruited individuals were divided into two cohorts according to the use of the spinal robotic system. All patients underwent conventional surgery before the robotic system was introduced in our institute in 2018. Patients in the RO group underwent surgery with the assistance of Mazor Robotics Renaissance™ (Medtronic, Denver, CO, USA), whereas those in the FH group underwent conventional FH pedicle screw placement. The process of installation and implementation has been explained in detail in a previous article (13).

All surgeries were performed via an open approach with a midline incision. Three brands of pedicle screws were used: Xia™ (Stryker, Kalamazoo, MI, USA), GZ Spinal Fixation System™ (Yi Hua Medical, Taichung, Taiwan), and Wiltrom Spinal Fixation System™ (Wiltrom Medical, Hsinchu, Taiwan). Screw diameters varied from 4.5–6.5 mm. Fusion bed preparation and bone grafting procedures were identical in both groups. We performed posterior column osteotomies (PCOs) at the apex of the scoliosis curve to achieve better correction of the deformity. Thorough decortication of the bilateral lamina was also performed at every level. Local bone chips, along with Bicera™ bone graft substitute (Wiltrom Medical, Hsinchu, Taiwan), were utilized for bone grafting. Lastly, the derotation technique was adapted for reduction of the spinal deformity.

### 2.2 Radiographic parameters

**Figure 1:**
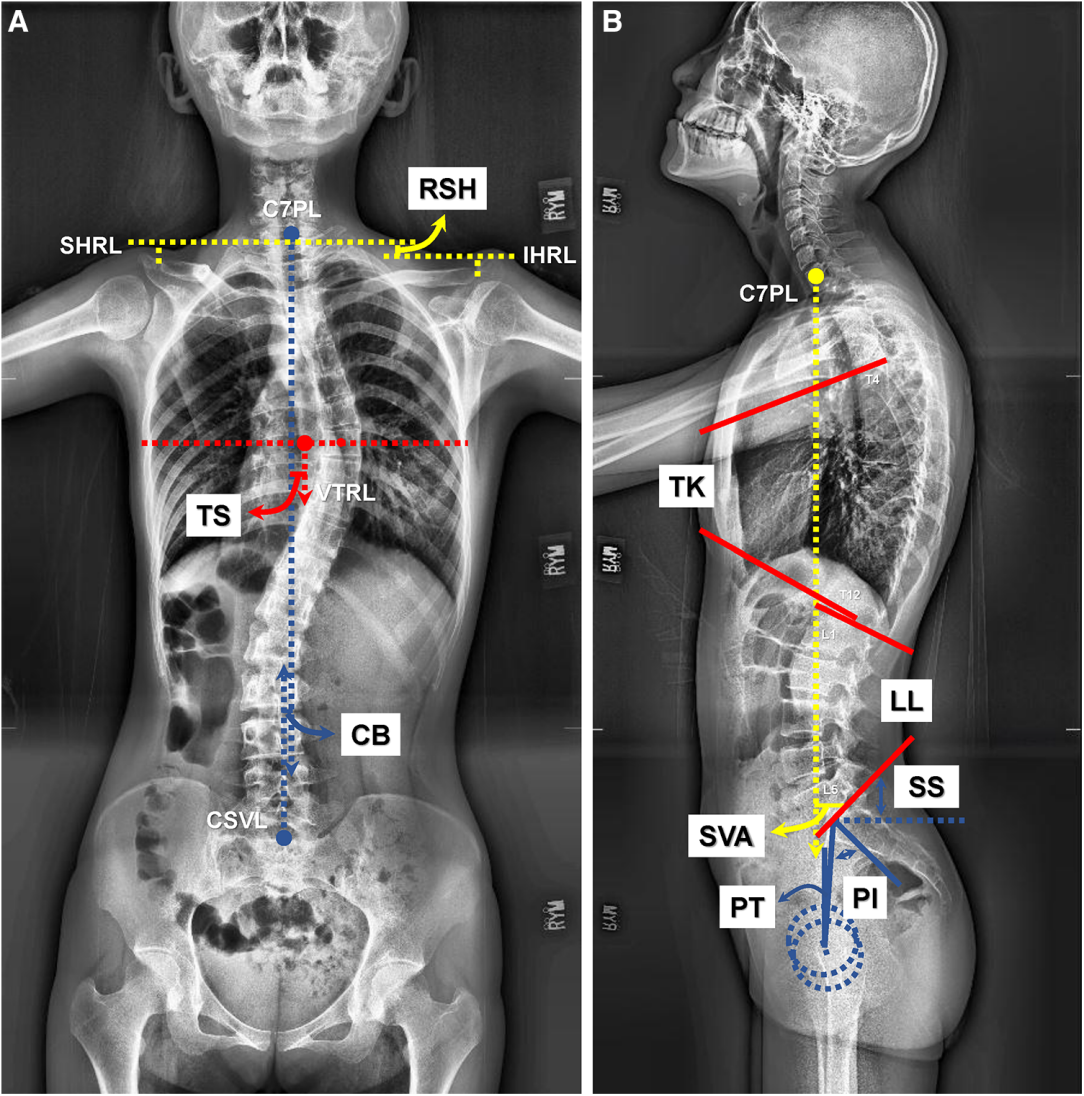
Illustration of the coronal and sagittal parameters on plain films. (**A**) Methods of measurement of the coronal parameters including coronal balance (CB), trunk shift (TS), and radiographic shoulder height (RSH) on plain anteroposterior radiograph. (**B**) Methods of measurement of the sagittal parameters including thoracic kyphosis (TK), lumbar lordosis (LL), sagittal vertical axis (SVA), pelvic tilt (PT), pelvic incidence (PI), and sacral slope (SS) on plain lateral radiograph.

Radiographic parameters observed in both coronal and sagittal radiographs included the following: Cobb's angle, coronal balance, trunk shift (TS), radiographic shoulder height (RSH), thoracic kyphosis, lumbar lordosis (LL), sagittal vertical axis (SVA), pelvic tilt (PT), pelvic incidence (PI), and sacral slope (SS), which were defined according to the Spinal Deformity Study Group manual (14) and measured using Surgimap™ software (Nemaris, New York, NY, USA). Screw density was defined as the total number of pedicle screws implanted per vertebra (15). The correction rate (CR) was calculated using the following formula: (preoperative Cobb's angle − postoperative Cobb's angle)/(preoperative Cobb's angle) × 100% (15). Normative data of the sagittal alignment parameters were extracted from the studies of Yukawa et al. (16) and Zhou et al. (17) in light of the geographical proximity and ethnic similarity, whereas coronal parameters were extracted from the study by Clement et al. (18). All definitions and normative data of these parameters are shown in Table 1 and illustrated in Figure 1. Additionally, the breech rate of the pedicle screws were evaluated on postoperative computed tomography (CT) by Gertzbein and Robbins' classification (19) and further defined as satisfactory (grade A or B) or unsatisfactory (grade C, D, or E) (20).

**Table 1:** Definitions and normative data of radiographic parameters.

Parameters	Definitions	Normative data (mean ± SD)
CB	Alignment of C7PL in relation to CSVL. Positive value if C7PL is on the right side.	−4 ± 12 mm (18)
TS	Horizontal distance between VTRL and CSVL. A trunk shift to the right of the CSVL is a positive value.	NA
RSH	Vertical distance between SHRL and IHRL. Positive value if the right shoulder is up.	5 ± 10 mm (18)
TK	Angle between T4 superior endplate and T12 inferior endplate.	41.8° ± 11.1° (17)
LL	Angle between L1 superior endplate and S1 superior endplate.	52.4° ± 13.1° (16)
SVA	Alignment of C7PL in relation to the posterior-superior corner of S1. Positive value if C7PL lies anteriorly.	−4.6 ± 13.5 mm (16)
PT	Angle between a line originating from center of femoral head to midpoint of sacral endplate and VRL. Positive value if VRL lies anteriorly.	11.4° ± 6.6° (16)
PI	Angle between a line originating from center of femoral head to midpoint of sacral endplate and a line perpendicular to center of sacral endplate.	51.8° ± 11.7° (16)
SS	Angle between S1 superior endplate and HRL.	40.3° ± 9.1° (16)

CB, coronal balance; TS, trunk shift; RSH, radiographic shoulder height; TK, thoracic kyphosis; LL, lumbar lordosis; SVA, sagittal vertical axis; PT, pelvic tilt; PI, pelvic incidence; SS, sacral slope; C7PL, C7 plumbline; CSVL, center sacral vertical line; VTRL, vertical trunk reference line; SHRL, superior horizontal reference line; IHRL, inferior horizontal reference line; VRL, vertical reference line; HRL, horizontal reference line; NA, not available; SD, standard deviation.

### 2.3 Clinical outcome measures

Clinical outcome measures included the visual analog scale (VAS) score for back pain, Oswestry Disability Index (ODI), and quality-adjusted life years (QALYs) obtained at each follow-up appointment, including at 1, 3, 6, and 12 months postoperatively. Initially, the patients completed the EuroQol five-dimensions (EQ5D) questionnaire, which was further converted to QALYs using the Japanese value set published by Tsuchiya (21).

### 2.4 Statistical analysis

The Mann–Whitney *U*-test and chi-squared test were used for continuous variables and categorical variables, respectively. Data in the tables are presented as medians with interquartile ranges (IQRs) enclosed by square brackets for continuous variables, and frequency with percentage enclosed by parentheses for categorical variables. The difference of radiographic parameters between pre- and postoperative values were analyzed using the Wilcoxon signed-rank test. Statistical analysis was conducted by professional statisticians affiliated with our institution using the SPSS 25 software (IBM, Armonk, NY, USA). The significance level was set at *p *< 0.05.

## 3 Results

### 3.1 Patient demographics and perioperative details

In total, 50 patients (19, FH group; 31, RO group) were eligible, with an average age of 17.6 years, an average follow-up period of 60.2 months, and female predominance (40/50, 80.0%). The demographic characteristics and preoperative status of the enrolled patients are outlined in Table 2, and there were no significant differences, except for the follow-up period. The perioperative details are described in Table 3. The Wiltrom Spinal Fixation System™ was utilized for 88.0% of all participants, and the proportion of brands used by the two groups was identical, *p *= 0.661. The RO technique placed significantly more pedicle screws per patient (median: 21.0, IQR: 17.0–24.0) than the FH group (13.0, 8.0–15.0), *p *< 0.001, while vertebrae instrumented per patient were similar. Consequently, the RO group achieved significantly higher pedicle screw density (2.0, 1.8–2.0) than the FH group (1.1, 0.8–1.8), *p *< 0.001. The correction rates were 59.1% for the FH group and 61.5% for the RO group, *p *= 0.204. The operative time was significantly prolonged with the assistance of the robot, at 510.0 (403.0–607.0) min, compared to 375.0 (299.0–450.0) min for FH surgery, *p *= 0.005. Nonetheless, operative time per screw was not different, 25.0 min for the RO group and 23.4 min for the FH group, *p *= 0.662. Additionally, the estimated blood losses (EBL) were not statistically different: 1,200.0 (800.0–2,700.0) ml for the RO group and 800.0 (700.0–1,700.0) ml for the FH group, *p *= 0.131. The EBL per screw were also similar, 70.6 and 72.7 ml for the RO and FH groups, respectively, *p *= 0.484. The average length of stay (LOS) was less for the RO group [7.0 (6.0–8.0) days] than that for the FH group [8.0 (7.0–9.0) days], although this was not statistically significant, *p *= 0.123.

**Table 2:** Overview of the demographic characteristics of the two groups.

	FH group (*n* = 19)		RO group (*n* = 31)		*p*-value
Age (years)	17.0	[15.0–19.0]	16.0	[14.0–21.0]	0.561
Sex					
Female, *n* (%)	14	(73.7%)	26	(83.9%)	0.474
BMI (kg/m^2^)	18.3	[15.9–19.1]	17.6	[16.8–21.1]	0.624
Curve type, *n* (%)					
1	4	(21.1%)	8	(25.8%)	0.106
2	1	(5.3%)	10	(32.3%)	
3	4	(21.1%)	3	(9.7%)	
4	3	(15.8%)	1	(3.2%)	
5	6	(31.6%)	9	(29.0%)	
6	1	(5.3%)	0	(0.0%)	
Preoperative Cobb's angle (°)	57.7	[44.8–76.8]	61.8	[47.8–75.3]	0.920
Preoperative VAS	1.0	[0.0–2.0]	3.0	[0.0–6.0]	0.216
Preoperative ODI	2.2	[0–15.6]	11.1	[0–15.6]	0.095
Preoperative QALYs	0.8	[0.7–1.0]	0.8	[0.5–1.0]	0.214
Follow-up (months)	98.0	[63.0–110.0]	37.0	[24.0–45.0]	<0.001*

FH, freehand; RO, robot-assisted; BMI, body mass index; Curve type: the Lenke classification system for AIS. VAS, visual analog scale; ODI, Oswestry disability index; QALYs, quality-adjusted life years. Mann–Whitney *U*-test was applied for continuous variables. Chi-squared test was applied for categorical variables. Data presentation: median [interquartile range]; frequency (percentage).

* Indicates *p* < 0.05.

**Table 3:** Perioperative details of the two groups.

	FH group		RO group		*p*-value
Implant brands, *n* (%)					
Stryker	3				0.661
Wiltrom	16	(15.8%)	28	(90.3%)	
GZ		(84.2%)	3	(9.7%)	
Screws inserted per patient	13.0	[8.0–15.0]	21.0	[17.0–24.0]	<0.001*
Vertebrae instrumented per patient	12.0	[8.0–13.0]	11.0	[10.0–13.0]	0.628
Screw density	1.1	[0.8–1.8]	2.0	[1.8–2.0]	<0.001*
Postoperative Cobb's angle (°)	22.8	[13.6–43.3]	21.8	[16.9–28.3]	0.332
Correction rate (%)	59.1	[48.5–71.6]	61.5	[58.9–72.7]	0.204
Operative time (min)	375.0	[299.0–450.0]	510.0	[403.0–607.0]	0.005*
Operative time per screw (min)	23.4	[17.4–34.0]	25.0	[22.3–28.8]	0.662
EBL (ml)	800.0	[700.0–1,700.0]	1,200.0	[800.0–2,700.0]	0.131
EBL per screw (ml)	72.7	[50.0–113.3]	70.6	[37.5–127.8]	0.484
LOS (days)	8.0	[7.0–9.0]	7.0	[6.0–8.0]	0.123

FH, freehand; RO, robot-assisted; EBL, estimated blood losses; LOS, length of stay. Mann–Whitney *U*-test for continuous variables. Chi-squared test for categorical variables. Data presentation: median [interquartile range]; frequency (percentage).

* Indicates *p* < 0.05.

### 3.2 Radiographic parameters

There were no significant differences in preoperative radiographic parameters between the two groups (Table 4). Table 5 further demonstrates the influence of surgical intervention on radiographic parameters, comparing both surgical methods. Patients in both groups had significantly improved Cobb's angles after corrective surgery. The RO technique provided a greater corrective force for TS (from 12.4 to −5.3 mm) and RSH (from 11.4 to −1.2 mm), both *p *= 0.001. The postoperative LL of both groups was within the normal range, while FH surgeries significantly reduced the LL from 56.0° to 47.4°, *p *= 0.013. FH surgery significantly changed the SVA, from negative to positive (−28.0–11.8 mm, *p *= 0.017). In contrast, the SVA in the RO group remained similar postoperatively. None of the three spinopelvic parameters, including PT, PI, and SS, differed postoperatively in either group. The breech rate of the pedicle screws was significantly lower for the RO group (9.5%) compared to the FH group (32.3%), *p *< 0.001 (Table 6).

**Table 4:** Preoperative radiographic parameters of the two groups.

	FH group		RO group		*p*-value
Cobb's angle (°)	57.7	[44.8–76.8]	61.8	[47.8–75.3]	0.920
CB (mm)	−9.9	[−13.8–11.8]	−1.5	[−15.5–13.8]	0.826
TS (mm)	7.2	[−20.9–20.1]	12.4	[−4.3–27.1]	0.194
RSH (mm)	10.4	[−7.1–22.4]	11.4	[2.6–21.9]	0.569
TK (°)	24.2	[8.5–40.9]	22.0	[14.7–33.7]	0.813
LL (°)	55.5	[46.4–67.9]	53.5	[40.3–59.6]	0.330
SVA (mm)	−28.0	[−40.2–10.5]	9.9	[−10.3–22.9]	0.071
PT (°)	5.1	[−0.7–15.2]	9.8	[1.2–13.9]	0.646
PI (°)	43.4	[36.8–55.5]	46.4	[38.4–52.7]	0.835
SS (°)	40.4	[36.1–44.3]	38.5	[31.1–46.0]	0.656

FH, freehand; RO, robot-assisted; CB, coronal balance; TS, trunk shift; RSH, radiographic shoulder height; TK, thoracic kyphosis; LL, lumbar lordosis; SVA, sagittal vertical axis; PT, pelvic tilt; PI, pelvic incidence; SS, sacral slope. Mann–Whitney *U*-test was performed. Data presentation: median [interquartile range].

**Table 5:** Comparison of the preoperative and postoperative radiographic parameters for the FH and RO groups.

	FH group					RO group				
	Preoperative		Postoperative		*p*-value	Preoperative		Postoperative		*p*-value
Cobb's angle (°)	57.7	[44.8–76.8]	22.8	[13.6–43.3]	<0.001*	61.8	[47.8–75.3]	21.8	[16.9–28.3]	<0.001*
CB (mm)	−9.9	[−13.8–11.8]	−10.2	[−18.2 to −5.9]	0.092	−1.5	[−15.5–13.8]	−9.4	[−17.3–3.8]	0.210
TS (mm)	7.2	[−20.9–20.1]	−7.4	[−18.6 to −2.8]	0.103	12.4	[−4.3–27.1]	−5.3	[−13.1–6.3]	0.001*
RSH (mm)	10.4	[−7.1–22.4]	0.0	[−6.2–10.3]	0.198	11.4	[2.6–21.9]	−1.2	[−8.9–10.6]	0.001*
TK (°)	24.2	[12.8–45.9]	31.3	[14.1–41.2]	0.575	22.1	[14.7–33.7]	20.0	[13.5–23.8]	0.118
LL (°)	56.0	[49.1–71.9]	47.4	[40.8–51.9]	0.013*	53.5	[40.3–59.6]	48.8	[37.9–53.1]	0.104
SVA (mm)	−28.0	[−40.2–10.5]	11.8	[−1.9–29.1]	0.017*	3.9	[−9.3–22.9]	5.5	[−16.5–16.1]	0.447
PT (°)	4.1	[−2.4–14.9]	2.3	[−1.3–20.7]	0.333	6.8	[−2.1–11.3]	7.9	[−0.6–18.0]	0.119
PI (°)	43.3	[36.3–56.1]	39.0	[36.5–53.0]	0.262	43.3	[34.9–49.5]	42.3	[35.9–51.8]	1.000
SS (°)	41.4	[35.2–46.3]	38.1	[32.3–39.2]	0.066	38.5	[25.6–45.5]	37.4	[28.4–41.7]	0.256

FH, freehand; RO, robot-assisted; CB, coronal balance; TS, trunk shift; RSH, radiographic shoulder height; TK, thoracic kyphosis; LL, lumbar lordosis; SVA, sagittal vertical axis; PT, pelvic tilt; PI, pelvic incidence; SS, sacral slope. Wilcoxon signed-rank test was performed. Data presentation: median [interquartile range].

* Indicates *p* < 0.05 between preoperative and postoperative values within each group.

**Table 6:** Breech rates of the pedicle screws of the two groups.

	FH group (*n *= 127)	RO group (*n *= 497)	*p*-value
Satisfactory, *n* (%)	86 (67.7%)	450 (90.5%)	<0.001*
Unsatisfactory, *n* (%)	41 (32.3%)	47 (9.5%)	

FH, freehand; RO, robot-assisted; Satisfactory: Gertzbein and Robbins classification grade A or B; Unsatisfactory: Gertzbein and Robbins classification grade C, D, or E. Chi-squared test was performed. Data presentation: frequency (percentage).

* Indicates *p* < 0.05.

### 3.3 Clinical outcome measures

**Figure 2:**
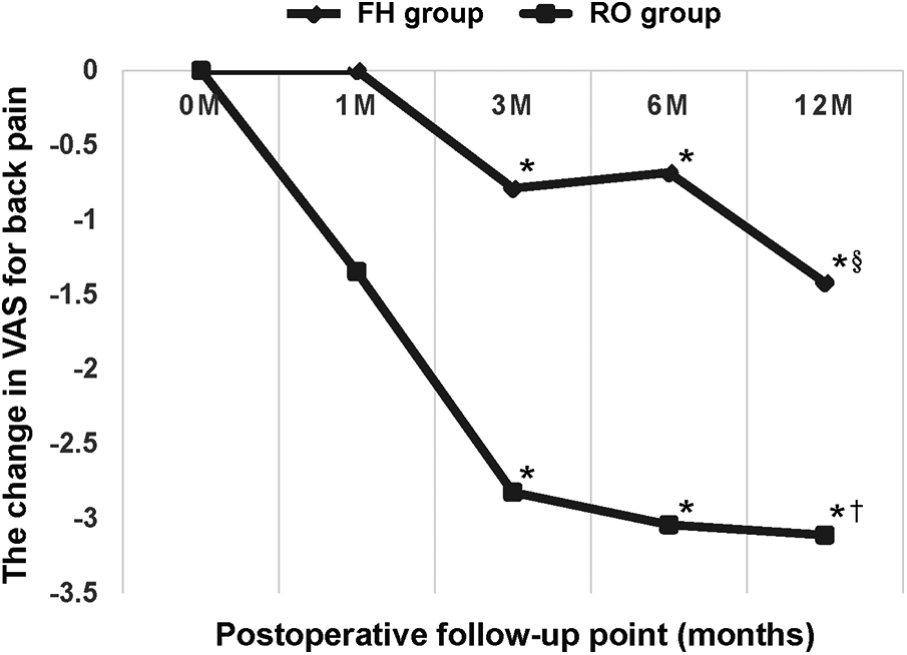
This line graph represents the change in visual analog score (VAS) score for back pain of the two groups at each follow-up point compared with preoperative value. The robot-assisted (RO) technique significantly outperformed conventional freehand (FH) surgery from the third postoperative month. * Indicates *p *< 0.05 between the two groups. § Indicates the significant improvement of ΔVAS_12m_ in the FH group compared with preoperative value (*p *< 0.05). † Indicates the significant improvement of ΔVAS_12m_ in the RO group compared with preoperative value (*p *< 0.05).

**Figure 3:**
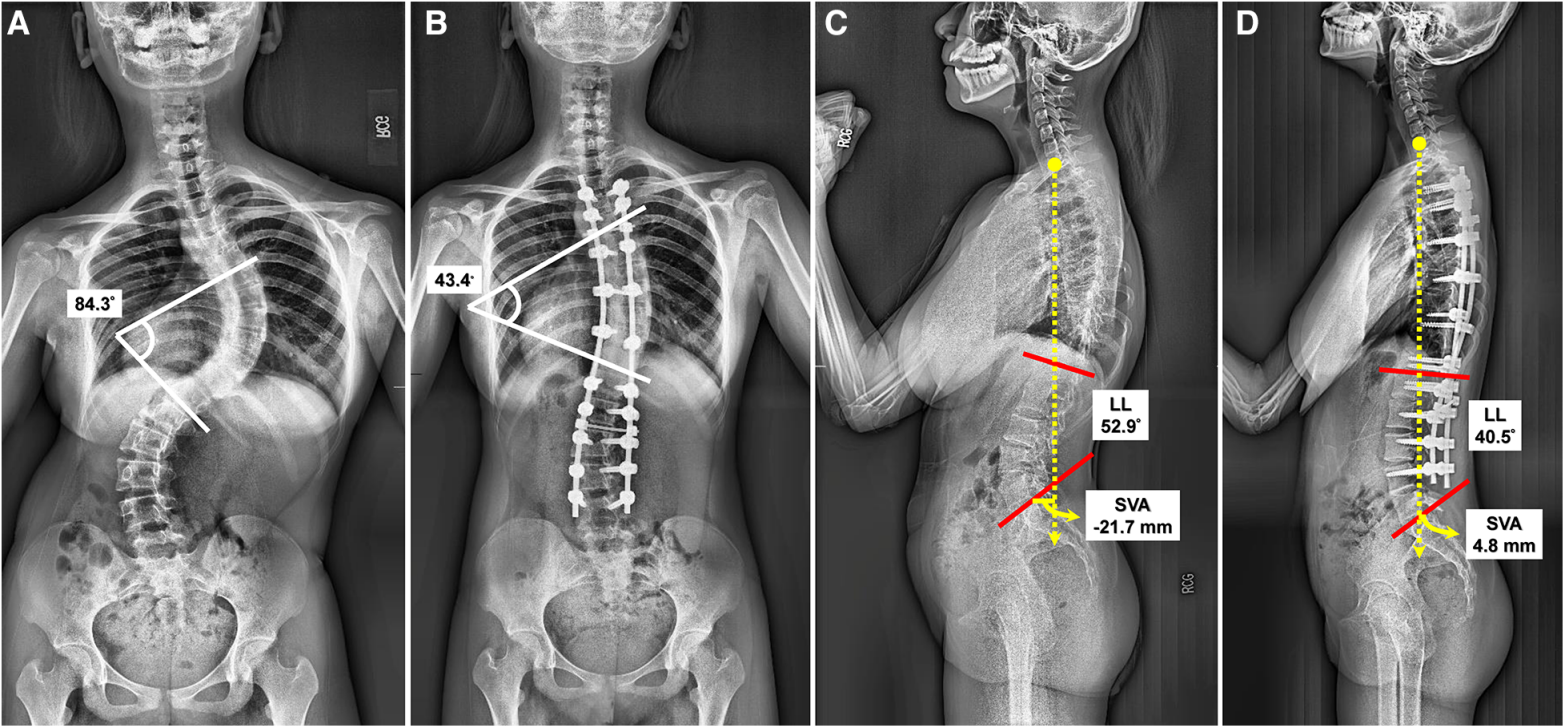
A female classified as lenke 3CN received freehand surgery at the age of 14 years. (**A**) The preoperative anteroposterior (AP) radiograph revealed a Cobb's angle of 84.3°. (**B**) The postoperative AP radiograph revealed a Cobb's angle of 43.4°, and correction rate (CR): 48.52%. The screw density was 1.43. (**C**) The preoperative lateral view showed lumbar lordosis (LL): 52.9°, and sagittal vertical axis (SVA): −21.7 mm. (**D**) The postoperative lateral view showed reduced LL (40.5°), and anteriorly moved SVA (4.8 mm).

**Figure 4:**
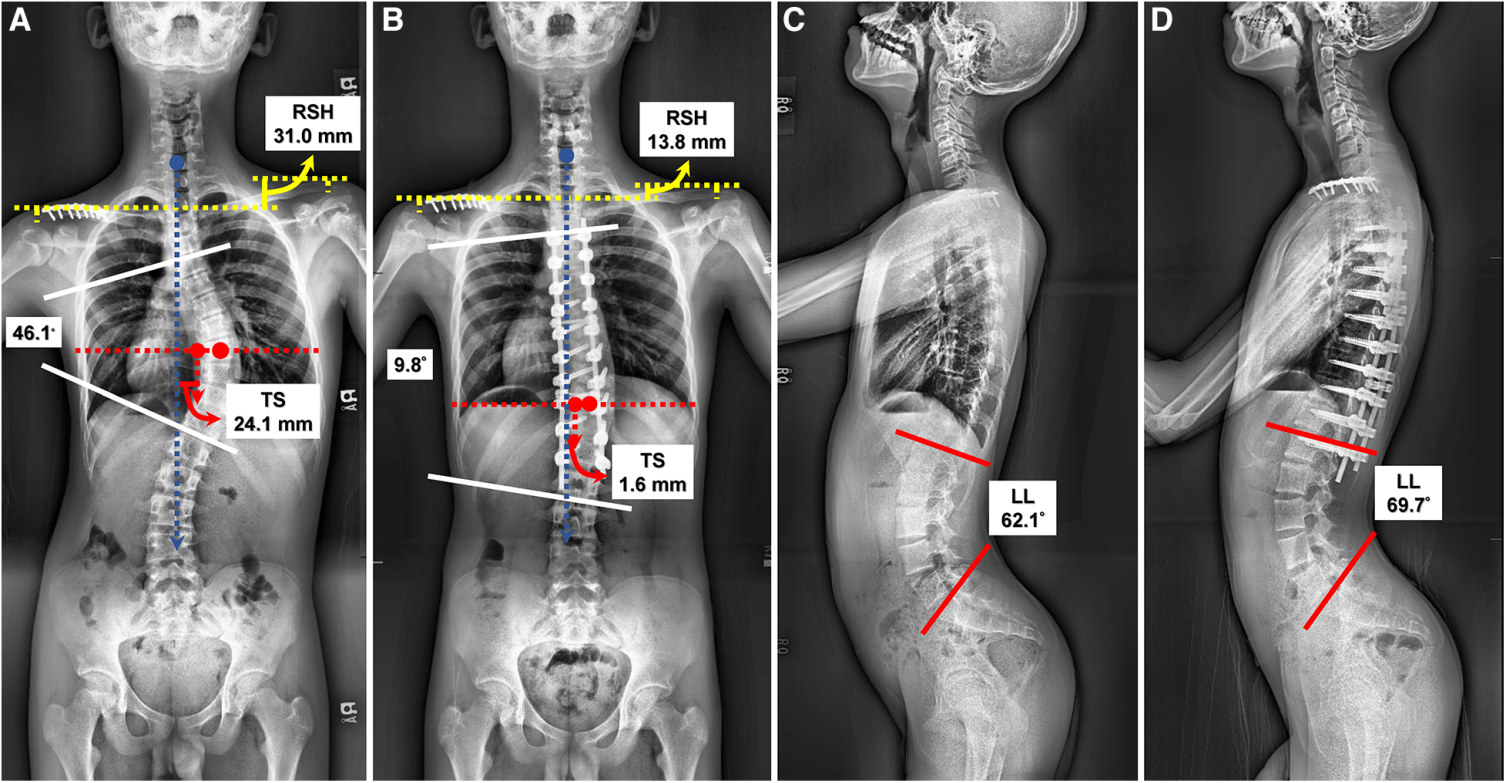
Another 14-year-old male with Lenke 1A- scoliosis underwent robot-assisted surgery. (**A**) The preoperative anteroposterior (AP) radiograph revealed a Cobb's angle of 46.1°, radiographic shoulder height (RSH): 31.0 mm, and trunk shift (TS): 24.1 mm. (**B**) The postoperative AP radiograph revealed a Cobb's angle of 9.8°, correction rate (CR): 78.74%, improved RSH (13.8 mm) and TS (1.6 mm). The screw density was 2.00. (**C**) The preoperative lateral view showed lumbar lordosis (LL): 62.1°. (**D**) The postoperative lateral view showed preserved LL (69.7°).

All three clinical outcome measures (VAS score for back pain, ODI, and QALYs) were similar preoperatively between the two cohorts as shown in Table 2. Table 7 further compares clinical outcome measures between groups preoperatively and at each follow-up point. Postoperative changes in the ODI and QALYs were not significantly different between the two groups, except for at the third month postoperatively (ΔODI_3m_, *p *= 0.031). The patients who underwent RO surgery experienced improved VAS scores from the third month postoperatively (ΔVAS_3m_), *p *= 0.017, as compared to those in the FH group, until the last follow-up one year after surgery (ΔVAS_12m_). Figure 2 demonstrates the trend in VAS changes. Postoperative ΔVAS_12m_ in both groups was significantly improved from preoperative values. To compare the radiographic outcomes of the two surgical techniques, we present one case each for the two cohorts (Figures 3, 4).

**Table 7:** Median changes (Δ) of clinical outcome measures of the two groups.

VAS	ΔVAS_1m_	ΔVAS_3m_	ΔVAS_6m_	ΔVAS_12m_
FH group	0 [−1.0–2.0]	0 [−2.0–1.0]	0 [−2.0–1.0]	0 [−2.0–0]
RO group	−1.0 [−4.0–1.0]	−3.0 [−5.5–0]	−3.0 [−6.0–0]	−3.0 [−6.0–0]
*p-*value	0.053	0.017*	0.011*	0.040*
ODI	ΔODI_1m_	ΔODI_3m_	ΔODI_6m_	ΔODI_12m_
FH group	22.2 [6.7–26.7]	8.9 [−2.2–15.6]	4.4 [−2.2–11.1]	0 [−6.7–6.7]
RO group	13.3 [4.5–22.2]	−2.2 [−14.5–8.9]	−2.2 [−22.2–8.9]	−12.2 [−25.6–6.1]
*p-*value	0.272	0.031*	0.100	0.308
QALYs	ΔQALYs_1m_	ΔQALYs_3m_	ΔQALYs_6m_	ΔQALYs_12m_
FH group	−0.06 [−0.19–0.03]	0 [0–0.21]	0.02 [0–0.27]	0.19 [0–0.27]
RO group	−0.07 [−0.28–0.18]	0.06 [0–0.33]	0.23 [0–0.51]	0.17 [0–0.54]
*p-*value	0.775	0.181	0.167	0.371

FH, freehand; RO, robot-assisted; VAS, visual analog scale; ODI, Oswestry disability index; QALYs, quality-adjusted life years; ΔVAS_1m_, the change in VAS for back pain between the first month postoperatively and preoperative value; ΔVAS_3m_, the 3rd month; ΔVAS_6m_, the 6th month; ΔVAS_12m_, the 12th month; ΔODI_1m_, the change in ODI between the first month postoperatively and preoperative value; ΔODI_3m_, the 3rd month; ΔODI_6m_, the 6th month; ΔODI_12m_, the 12th month; ΔQALYs_1m_, the change in QALYs between the first month postoperatively and preoperative value; ΔQALYs_3m_, the 3rd month; ΔQALYs_6m_ the 6th month; ΔQALYs_12m_, the 12th month. Mann–Whitney *U*-test was performed. Data presentation: median [interquartile range].

* Indicates *p* < 0.05 between the two groups.

## 4 Discussion

The RO technique may outperformed the conventional FH posterior instrumentation surgery for AIS patients in certain aspects. However, the follow-up period was significantly shorter in the RO group, as the Renaissance™ robotic system was not introduced in our institute until 2018. A higher pedicle screw density was attained with RO compared to that with the FH method. The correction rates were similar, at approximately 60%. The operative time per screw and EBL per screw did not differ between the two surgical methods.

Both groups underwent an open approach with a midline incision, with similar LOS in both groups; this correlates with the findings by Schatlo et al. (22) that LOS was not statistically different between RO and conventional FH open-approach techniques (9.8 days vs. 10.3 days), *p *= 0.390. Hyun et al. (23) compared robot-guided minimally invasive surgery and fluoroscopic-guided open surgery, with LOS reported to be 6.8 days vs. 9.4 days, *p *= 0.020. It can be concluded that the surgical approach, open or minimally invasive, independently affects LOS, regardless of robotic use (9, 23).

A retrospective multicenter study of postoperative TS in patients with AIS deemed a horizontal deviation greater than 2 cm of the vertical trunk reference line (VTRL) from the center sacral vertical line (CSVL) post-surgically as positive TS. The prevalence of positive TS was found to reduce from 29.3% to 13.6% after surgical intervention (24), similar to the results of our study (from 48.0% to 8.0%). We further conducted the McNemar test to analyze the change in positive TS postoperatively using two different techniques. The TS for the RO group was significantly reduced from 45.1% to 3.2%, *p *< 0.001, a larger reduction than that for the FH group (52.7%–15.8%), *p *= 0.065. Patient without trunk shift preoperatively who developed trunk shift after the surgery was considered iatrogenic. In the FH group, two patients (10.5%) exhibited iatrogenic trunk shift, while in the RO group, there were no instances (0%). The RO technique provides more effective correction for TS, and also reduces the risk of iatrogenic trunk shift compared to the FH method.

The existing literature primarily focuses on using RSH as a parameter to predict postoperative shoulder imbalance (PSI). Unbalanced shoulders are defined as having an RSH of 10 mm or more (25). Studies have identified preoperative RSH as an independent predictor of PSI (26). Our data suggest that shoulder imbalance shows significant improvement after RO surgery, in contrast to the FH group, where RSH remains relatively unchanged. We recommend considering RO surgery for patients with preoperative shoulder imbalance to achieve better RSH correction and thereby reduce the risk of PSI.

Due to the financial limitations imposed by our National Health Insurance system, titanium rod remains the sole option for posterior spinal instrumentation in our healthcare setting. Previous literature suggests that cobalt-chromium rods are generally considered better than titanium rods for effectively reducing the rate of rod fractures, correcting spinal deformities, and ensuring postoperative stability (27, 28). Limited to using titanium rods, we need to increase pedicle screw density to effectively and safely perform the reduction by derotation technique with the rods. Through preoperative planning and the assistance of a robot during surgery, surgeons are able to implant a greater number of pedicle screws, achieving a higher screw density.

Postoperative plain radiographs demonstrated significantly reduced LL values for patients in the FH group, whereas RO surgery maintained the LL. Iatrogenic loss of LL is a disabling complication after corrective scoliosis surgery, resulting in the inability to stand upright and back pain (29). Chun et al. (30) also reported a strong relationship between low back pain (LBP) and lumbar lordotic angle. Given this evidence, we concluded that the RO technique could provide sustained postoperative LL, resulting in less LBP than the conventional FH method. This corresponded with our finding of significantly improved back pain three months postoperatively (ΔVAS_3m_) with the use of a robot. We suggest that special attention should be paid while operating on patients with especially small LL using the traditional FH method.

We observed that SVA was significantly increased, moving anteriorly, after conventional FH surgery, but remained unchanged in the RO group. The postoperative SVAs for both groups were within the physiological range of the child (−31.6–22.4 mm). Notably, postoperative SVA in the FH group was beyond the normal range (9.5 mm) of the adult Schwab Adult Spinal Deformity Classification (31). The RO technique may be better for sustaining patient SVA.

As for the accuracy of RO pedicle screw instrumentation, previous studies have already verified its accuracy, ranging from 90% to 100% (32). In our study, the rate of satisfactory screw position was 90.5%; we believe that this is related to the pedicle hypoplasia commonly present in AIS patients. Additionally, we aimed to achieve a higher screw density when performing surgery. Provided that the screw could reach the vertebral body during preoperative planning, we could specially design an “in-out-in” trajectory for some patients with pedicle hypoplasia. To avoid medial wall violation, it is feasible to increase the total number of pedicle screws in a safe way (only lateral breech), and further achieve better corrective force. Conversely, such a trajectory could not be designed preoperatively if using the conventional FH method, and we would rather not insert screws at the vertebrae of patients with pedicle hypoplasia.

Our data demonstrates that the RO group had a significantly lower breach rate compared to the FH group (9.5% vs. 32.3%, *p *< 0.001). This reduced breach rate allows for the successful placement of a larger number of pedicle screws, significantly increasing screw density. Hwang et al. have reported that high-density pedicle screw constructs lead to better deformity correction in AIS patients (33). The RO technique, by achieving higher screw density, provides stronger spinal fixation, enabling surgeons to safely perform more effective deformity corrections.

The changes in the ODI and QALYs after surgery were not significantly different between the two groups, except for ΔODI_3m_. However, all patients reported better function and quality of life 12 months postoperatively compared with that observed preoperatively. The VAS for back pain was the main clinical parameter that verified the superiority of the RO surgery. The RO technique outperformed conventional FH surgery at alleviating LBP from the third postoperative month (ΔVAS_3m_). This correlated with the radiographic finding that RO surgery could maintain postoperative LL, resulting in reduced LBP compared with that with the conventional FH method.

Costa et al. (34) performed a biomechanical study and found that a misplaced screw in the craniocaudal direction was associated with significantly less primary stability than screws in the centered sagittal position. Açikbaş et al. (35) also found that significant spinal motion on flexion-extension radiographs was observed in patients with screw misplacement, and this significant motion was correlated with more intense back pain. Robotic technology has demonstrated significantly superior accuracy with fewer misplaced pedicle screws compared to that with conventional techniques (8, 11, 12), providing stability to the spinal structures and further alleviating back pain.

To our knowledge, this study is the first to highlight the radiographic and clinical outcomes of RO pedicle screw instrumentation in patients with AIS; this is clinically important in assisting surgeons with adopting RO techniques.

## 5 Limitations

First, the sample size was small, which could be owing to the inclusion of only all-pedicle-screw construct and exclusion of hybrid implants. Second, there is chronological bias. Before the robotic system was first introduced in our institute in 2018, patients could only choose conventional FH surgery, which caused a significant difference in follow-up time between the two groups. Finally, data from only one institute were included.

## 6 Conclusions

Overall, RO pedicle screw instrumentation achieves a significantly reduced breech rate and provides satisfactory radiographic and clinical outcomes in AIS patients. TS and RSH were significantly corrected with preserved LL, resulting in an improved VAS score for back pain.

## Data Availability

The raw data supporting the conclusions of this article will be made available by the authors, without undue reservation.

## References

[1] Horne JP Flannery R Usman S. Adolescent idiopathic scoliosis: diagnosis and management. Am Fam Phys. (2014) 89:193–8. 24506121

[2] Weinstein SL Dolan LA Cheng JCY Danielsson A Morcuende JA. Adolescent idiopathic scoliosis. Lancet. (2008) 371:1527–37. 10.1016/S0140-6736(08)60658-3 18456103

[3] Lonstein JE Carlson JM. The prediction of curve progression in untreated idiopathic scoliosis during growth. J Bone Joint Surg Am. (1984) 66:1061–71. 10.2106/00004623-198466070-00013 6480635

[4] Suk SI Lee CK Kim WJ Chung YJ Park YB. Segmental pedicle screw fixation in the treatment of thoracic idiopathic scoliosis. Spine (Phila Pa 1976). (1995) 20:1399–405. 10.1097/00007632-199506020-00012 7676339

[5] Crostelli M Mazza O Mariani M. Free-hand pedicle screws insertion technique in the treatment of 120 consecutive scoliosis cases operated without use of intraoperative neurophysiological monitoring. Eur Spine J. (2012) 21:43–9. 10.1007/s00586-012-2218-y PMC3325392 22411036

[6] Crostelli M Mazza O Mariani M Mascello D. Treatment of severe scoliosis with posterior-only approach arthrodesis and all-pedicle screw instrumentation. Eur Spine J. (2013) 22:808–14. 10.1007/s00586-013-3027-7 PMC3830024 24061974

[7] Laudato PA Pierzchala K Schizas C. Pedicle screw insertion accuracy using o-arm, robotic guidance, or freehand technique: a comparative study. Spine. (2018) 43:E373–8. 10.1097/BRS.0000000000002449 29019807

[8] Fatima N Massaad E Hadzipasic M Shankar GM Shin JH. Safety and accuracy of robot-assisted placement of pedicle screws compared to conventional free-hand technique: a systematic review and meta-analysis. Spine J. (2021) 21:181–92. 10.1016/j.spinee.2020.09.007 32976997

[9] Ghasem A Sharma A Greif DN Alam M Maaieh MA. The arrival of robotics in spine surgery: a review of the literature. Spine (Phila Pa 1976). (2018) 43:1670–7. 10.1097/BRS.0000000000002695 29672420

[10] D’Souza M Gendreau J Feng A Kim LH Ho AL Veeravagu A. Robotic-assisted spine surgery: history, efficacy, cost, and future trends. Robot Surg. (2019) 6:9–23. 10.2147/RSRR.S190720 31807602 PMC6844237

[11] Joseph JR Smith BW Liu X Park P. Current applications of robotics in spine surgery: a systematic review of the literature. Neurosurg Focus. (2017) 42:E2. 10.3171/2017.2.FOCUS16544 28463618

[12] Li HM Zhang RJ Shen CL. Accuracy of pedicle screw placement and clinical outcomes of robot-assisted technique versus conventional freehand technique in spine surgery from nine randomized controlled trials: a meta-analysis. Spine (Phila Pa 1976). (2020) 45:E111–9. 10.1097/BRS.0000000000003193 31404053

[13] Lai YP Lin YH Wu YC Shih CM Chen KH Lee CH Robot-assisted pedicle screw placement led to lower screw loosening rate than fluoroscopy-guided technique in transforaminal lumbar interbody fusion for lumbar degenerative disease: a single-center retrospective study. J Clin Med. (2022) 11:4989. 10.3390/jcm11174989 36078918 PMC9456711

[14] O’Brien MF Kuklo TR Blanke KM Lenke LG. Spinal Deformity Study Group Radiographic Measurement Manual. Memphis, Tenn.: Medtronic Sofamor Danek USA Inc. (2008).

[15] Quan GM Gibson MJ. Correction of main thoracic adolescent idiopathic scoliosis using pedicle screw instrumentation: does higher implant density improve correction? Spine (Phila Pa 1976). (2010) 35:562–7. 10.1097/BRS.0b013e3181b4af34 20118842

[16] Yukawa Y Kato F Suda K Yamagata M Ueta T Yoshida M. Normative data for parameters of sagittal spinal alignment in healthy subjects: an analysis of gender specific differences and changes with aging in 626 asymptomatic individuals. Eur Spine J. (2018) 27:426–32. 10.1007/s00586-016-4807-7 27771788

[17] Zhou XY Zhao J Li B Wang ZB Zhang ZC Hu W Assessment of sagittal spinopelvic balance in a population of normal Chinese children. Spine (Phila Pa 1976). (2020) 45:E787–91. 10.1097/BRS.0000000000003428 32049939

[18] Clement RC Anari J Bartley CE Bastrom TP Shah R Talwar D What are normal radiographic spine and shoulder balance parameters among adolescent patients? Spine Deform. (2020) 8:621–7. 10.1007/s43390-020-00074-9 32096131

[19] Gertzbein SD Robbins SE. Accuracy of pedicular screw placement in vivo. Spine (Phila Pa 1976). (1990) 15:11–4. 10.1097/00007632-199001000-00004 2326693

[20] Zhang JN Fan Y Hao DJ. Risk factors for robot-assisted spinal pedicle screw malposition. Sci Rep. (2019) 9:3025. 10.1038/s41598-019-40057-z 30816334 PMC6395613

[21] Tsuchiya A Ikeda S Ikegami N Nishimura S Sakai I Fukuda T Estimating an EQ-5D population value set: the case of Japan. Health Econ. (2002) 11:341–53. 10.1002/hec.673 12007165

[22] Schatlo B Molliqaj G Cuvinciuc V Kotowski M Schaller K Tessitore E. Safety and accuracy of robot-assisted versus fluoroscopy-guided pedicle screw insertion for degenerative diseases of the lumbar spine: a matched cohort comparison. J Neurosurg Spine. (2014) 20:636–43. 10.3171/2014.3.SPINE13714 24725180

[23] Hyun SJ Kim KJ Jahng TA Kim HJ. Minimally invasive robotic versus open fluoroscopic-guided spinal instrumented fusions: a randomized controlled trial. Spine (Phila Pa 1976). (2017) 42:353–8. 10.1097/BRS.0000000000001778 27398897

[24] Trobisch PD Samdani AF Pahys JM Cahill PJ. Postoperative trunk shift in Lenke 1 and 2 curves: how common is it? And analysis of risk factors. Eur Spine J. (2011) 20:1137–40. 10.1007/s00586-011-1820-8 21533598 PMC3176686

[25] Han X Liu Z Qiu Y Sha S Yan H Jin M Clavicle chest cage angle difference: is it a radiographic and clinical predictor of postoperative shoulder imbalance in Lenke I adolescent idiopathic scoliosis? Spine (Phila Pa 1976). (2016) 41:1346–54. 10.1097/BRS.0000000000001521 26909841

[26] Zang L Fan N Hai Y Lu SB Su QJ Yang JC Evaluation of the predictors of postoperative aggravation of shoulder imbalance in severe and rigid thoracic or thoracolumbar scoliosis. Eur Spine J. (2016) 25:3353–65. 10.1007/s00586-015-4313-3 26538156

[27] Shinohara K Takigawa T Tanaka M Sugimoto Y Arataki S Yamane K Implant failure of titanium versus cobalt-chromium growing rods in early-onset scoliosis. Spine (Phila Pa 1976). (2016) 41:502–7. 10.1097/BRS.0000000000001267 26966974

[28] Shega FD Zhang HQ Manini DR Tang MX Liu SH. Comparison of effectiveness between cobalt chromium rods versus titanium rods for treatment of patients with spinal deformity: a systematic review and meta-analysis. Adv Orthop. (2020) 1:8475910. 10.1155/2020/8475910 PMC7491467 32963834

[29] La Grone MO. Loss of lumbar lordosis. A complication of spinal fusion for scoliosis. Orthop Clin North Am. (1988) 19:383–93. 10.1016/S0030-5898(20)30318-7 3282206

[30] Chun SW Lim CY Kim K Hwang J Chung SG. The relationships between low back pain and lumbar lordosis: a systematic review and meta-analysis. Spine J. (2017) 17:1180–91. 10.1016/j.spinee.2017.04.034 28476690

[31] Schwab F Ungar B Blondel B Buchowski J Coe J Deinlein D Scoliosis research society-schwab adult spinal deformity classification: a validation study. Spine (Phila Pa 1976). (2012) 37:1077–82. 10.1097/BRS.0b013e31823e15e2 22045006

[32] McKenzie DM Westrup AM O'Neal CM Lee BJ Shi HH Dunn IF Robotics in spine surgery: a systematic review. J Clin Neurosci. (2021) 89:1–7. 10.1016/j.jocn.2021.04.005 34119250

[33] Hwang CJ Baik JM Cho JH Yoon SJ Lee DH Lee CS. Posterior correction of adolescent idiopathic scoliosis with high-density pedicle screw-only constructs: 5 years of follow-up. Yonsei Med J. (2020) 61:323–30. 10.3349/ymj.2020.61.4.323 32233175 PMC7105406

[34] Costa F Villa T Anasetti F Tomei M Ortolina A Cardia A Primary stability of pedicle screws depends on the screw positioning and alignment. Spine J. (2013) 13:1934–9. 10.1016/j.spinee.2013.03.046 23684239

[35] Açikbaş SC Arslan FY Tuncer MR. The effect of transpedicular screw misplacement on late spinal stability. Acta Neurochir (Wien). (2003) 145:949–54; discussion 954. 10.1007/s00701-003-0116-0 14628199

